# Supplementary material for: The Mediterranean Diet in Pregnancy: Implications for Maternal Brain Morphometry in a Secondary Analysis of the IMPACT BCN Randomized Clinical Trial
Source: Nutrients. 2024 May 24;16(11):1604. doi: 10.3390/nu16111604 (PMC11174669; doi:10.3390/nu16111604)
Supplement: Supplementary file 1 [file nutrients-16-01604-s001.zip › nutrients-2990047-supplementary.pdf]

Supplementary Document

Contents

TABLE S1. 2

TABLE S2. 3

TABLE S3. 4

TABLE S4. 5

TABLE S5. 6

TABLE S6. 7

FIGURE S1. 8

Table S1. Food consumption assessed at the end of the intervention in each group by adjusting for the baseline values.

	**Mediterranean diet** n=34	**Usual Care** n=37	P^b	*Difference(95% CI)*
Extra Virgin Olive Oil – g/d	48.8 (2.62)	34.8 (2.58)	<0.001	13.9 (6.64 to 21.1)
Refined olive oil – g/d	1.47 (1.94)	6.41 (1.94)	0.072	-4.94 (-10.3 to 0.44)
Total nuts – g/d	38.6 (3.38)	21.6 (3.28)	<0.001	17.0 (7.76 to 26.2)
Vegetables – g/d	327.5 (23.5)	264.6 (23.1)	0.056	62.9 (-1.72 to 127.5)
Legumes – g/d	84.3 (8.44)	45.2 (8.19)	<0.001	39.1 (16.0 to 62.2)
Fruits – g/d	421.9 (50.3)	296.4 (49.5)	0.075	125.5 (-12.8 to 236.8)
Refined cereals – g/d	22.0 (7.14)	63.8 (7.04)	<0.001	-41.8 (-61.5 to 22.1)
Whole grain cereals – g/d	71.1 (5.94)	35.1 (5.85)	<0.001	36.0 (19.7 to 52.4)
Fish or seafood – g/d	99.0 (6.15)	74.6 (6.06)	0.005	24.4 (7.45 to 41.3)
Fatty fish – g/d	35.1 (3.81)	20.5 (3.76)	0.006	14.6 (4.12 to 25.1)
Lean meat – g/d	82.7 (6.74)	68.1 (6.64)	0.121	14.6 (-3.89 to 33.2)
Red meat – g/d	28.3 (4.46)	48.8 (4.39)	0.001	-20.5 (-32.7 to -8.21)
Processed meat – g/d	30.4 (3.70)	36.8 (3.65)	0.216	-6.43 (-16.6 to 3.75)
Pastries, cakes, or sweets – g/d	23.8 (5.11)	42.9 (4.97)	0.008	-19.0 (-33.0 to -5.06)
Dairy products – g/d	465.7 (36.8)	396.9 (35.7)	0.180	68.8 (-31.7 to 169.2)

Final values are expressed as means (SE) and comparison among groups done with ANCOVA analysis. [b]ANCOVA analysis.

Table S2. Energy and nutrient intake assessed at the end of the intervention in each group by adjusting for the baseline values.

	**Mediterranean diet** n=34	**Usual Care** n=37		
			P^b	*Difference (95% CI)*
Energy – kcal/d	2612 (80.3)	2467 (79.1)	0.199	145.0 (-76.0 to 366.0)
Protein – kcal/d	118.5 (4.29)	104.1 (4.23)	0.017	14.4 (2.59 to 26.2)
Carbohydrate – g/d	213.9 (10.0)	217.8 (9.90)	0.780	-3.93 (-31.6 to 23.7)
Fiber – g/d	40.2 (2.06)	31.4 (2.03)	0.002	8.77 (3.11 to 14.4)
Total fat – g/d	142.4 (4.29)	130.9 (4.22)	0.055	11.5 (-0.27 to 23.3)
SFA – g/d	35.5 (1.56)	36.0 (1.54)	0.850	-0.41 (-4.71 to 3.88)
MUFA – g/d	67.0 (2.11)	62.0 (2.08)	0.095	4.95 (-0.87 to 10.8)
PUFA – g/d	29.3 (1.22)	23.4 (1.20)	<0.001	5.85 (2.49 to 9.20)
α-Linoleic acid – g/d	19.3 (0.93)	15.8 (0.92)	0.006	3.57 (1.00 to 6.13)
α-Linolenic acid – g/d	2.54 (0.10)	1.40 (0.10)	<0.001	1.13 (0.85 to 1.42)
EPA – g/d	0.27 (0.02)	0.18 (0.02)	0.002	0.09 (0.03 to 0.15)
DHA – g/d	0.62 (0.05)	0.40 (0.05)	0.004	0.22 (0.07 to 0.37)
Trans-FA – g/d	1.32 (0.18)	1.48 (0.17)	0.515	-0.16 (-0.65 to 0.33)
Cholesterol – mg/d	335.1 (15.9)	318.0 (15.7)	0.444	17.1 (-26.7 to 60.9)

SFA Saturated fatty acids, MUFA Monounsaturated fatty acids, PUFA Polyunsaturated fatty acids, DHA Docosahexaenoic acid, EPA Eicosapentaenoic acid.

Final values are expressed as means (SE) and comparison among groups done with ANCOVA analysis. [b]ANCOVA analysis.

Table S3. Mediterranean diet score at the final visit adjusted by baseline assessment.

		Mediterraenan diet<br>n=34	Usual care<br>n=37	*p*	
**Mediterranean diet score**					Adjusted mean difference (SE)
	Baseline[a]	8.06 (2.61)	7.76 (2.43)		
	Final[b]	12.61 (0.45)**	8.38 (0.43)	<0.001	4.23 (2.99 to 5.46)
**Improvements in the Mediterranean diet score**					
	High	24 (71%)	6 (16%)	<0.001	
	Low	10 (29%)	31 (84%)		

[a]Baseline values are observed as means (SD) or number (%).
[b]Final value are baseline-adjusted (least-square) means (SE) and comparison among groups done with linear regression analysis.
*p<0.05 and **p<0.001 final from baseline comparison

Table S4. Cortical surface area difference between Mediterranean diet group and usual care group participants.

Contrast	Regions	Hemisphere	MNI x	MNI y	MNI z	CWP	CWP low-high	Cluster size (mm2)
**Mediterranean diet > Usual care**	Superiorparietal	Left	-15.7	-64.6	61.3	<0.001	0.00000 – 0.00040	3270.69
	Precuneus	Right	6.7	-59.9	49.9	0.03	0.02642 – 0.03253	2380.19
**Usual care > Mediterranean diet**	-							

CWP: clusterwise p-value, CWPLow-High: 90% confidence interval for CWP, MNI: Tal (XYZ) is the Talairach (MNI305) coordinate of the maximum. Results are generated from a general linear model with total intracranial volume, age, magnetic resonance protocol and nulliparity.

Table S5. Cortical surface area differences between Mediterranean diet group participants and usual care group participants, with model 2 general linear model.

Contrast	Regions	Hemisphere	MNI x	MNI y	MNI z	CWP	CWP low-high	Cluster size (mm2)
**Mediterranean diet > Usual care**	Superiorparietal	Left	-9.7	-71.6	51.0	0.004	0.0032 – 0.00559	2800.85
	Precuneus	Right	6.0	-69.2	37.6	0.002	0.01931 – 0.02445	2501.2
**Usual care > Mediterranean diet**	-							

CWP: clusterwise p-value, CWPLow-High: 90% confidence interval for CWP , MNI: Tal(XYZ) is the Talairach (MNI305) coordinate of the maximum.
*Size indicates surface area of cluster in each brain area.
General linear model with total intracranial volume, age, MRI protocol and nulliparity, Mediterranean diet score at the initial assessment and socio-economic status.

Table S6. Summary of 17-item dietary assessment used to evaluate adherence to the Mediterranean diet.

Food	Criteria*
How many tablespoons of **extra virgin olive oil** do you consume per day (for cooking, dressing, at restaurants, etc.)?	≥4; 1 point
How many servings of **vegetables** do you consume per day? Count garnish and side serving as ½ point. 1 serving = 200g	≥3; 1 point
How many pieces of **fruit** do you consume per day?	≥2; 1 point
How many servings of cooked vegetables, pasta, rice or other dishes seasoned with ***sofrito*** (tomato, garlic, onion or leek sauce made with extra virgin olive oil and low heat) do you consume per week?	≥2; 1 point
How many servings of **whole grain cereals**, bread, pasta, or rice do you consume per week?	≥5; 1 point
How many servings **of refined cereals**, bread, pasta, or rice do you consume per week?	<3; 1 point
How many servings of **legumes** do you consume per week? 1 serving =150g	≥3; 1 point
How many servings of **fish/seafood** do you consume per week? 1 serving = 100-150g fish; 4-5 pieces or 200g of seafood.	≥3; 1 point
How many servings of **fatty fish** do you consume per week? 1 serving = 100-150g	≥1; 1 point
How many servings of **red meat**, including beef, lamb, non-lean pork, duck do you consume per week? 1 serving = 100-150g.	≤1; 1 point
How many servings of **processed meat**, including hamburgers or sausages do you consume per week? 1 serving = 100-150g	≤1; 1 point
How many servings of **chicken**, turkey, rabbit, or lean pork do you consume per week? 1 serving = 100-150g	≥3; 1 point
How many **carbonated and/or sugar-sweetened beverages** do you consume per week?	<1; 1 point
How many servings of **nuts**, including walnut, hazelnut, almond, peanut, or pistachio do you consume per week? 1 serving = 30g	≥3; 1 point
How many times do you consume **pastries** such as cookies, custard, sweets, or cake, per week?	<2; 1 point
How many servings of **dairy**, including milk, yogurt, cheese, or calcium-fortified vegetable milk do you consume per day?	≥3; 1 point
How many servings of **butter, margarine, or cream** do you consume per week?	<1; 1 point

*0 points if these criteria were not met.

Figure S1. Inflated brain images of the cortical surface area with significant differences in Mediterranean diet group participants compared to usual care group participants, with model 2 general linear model.

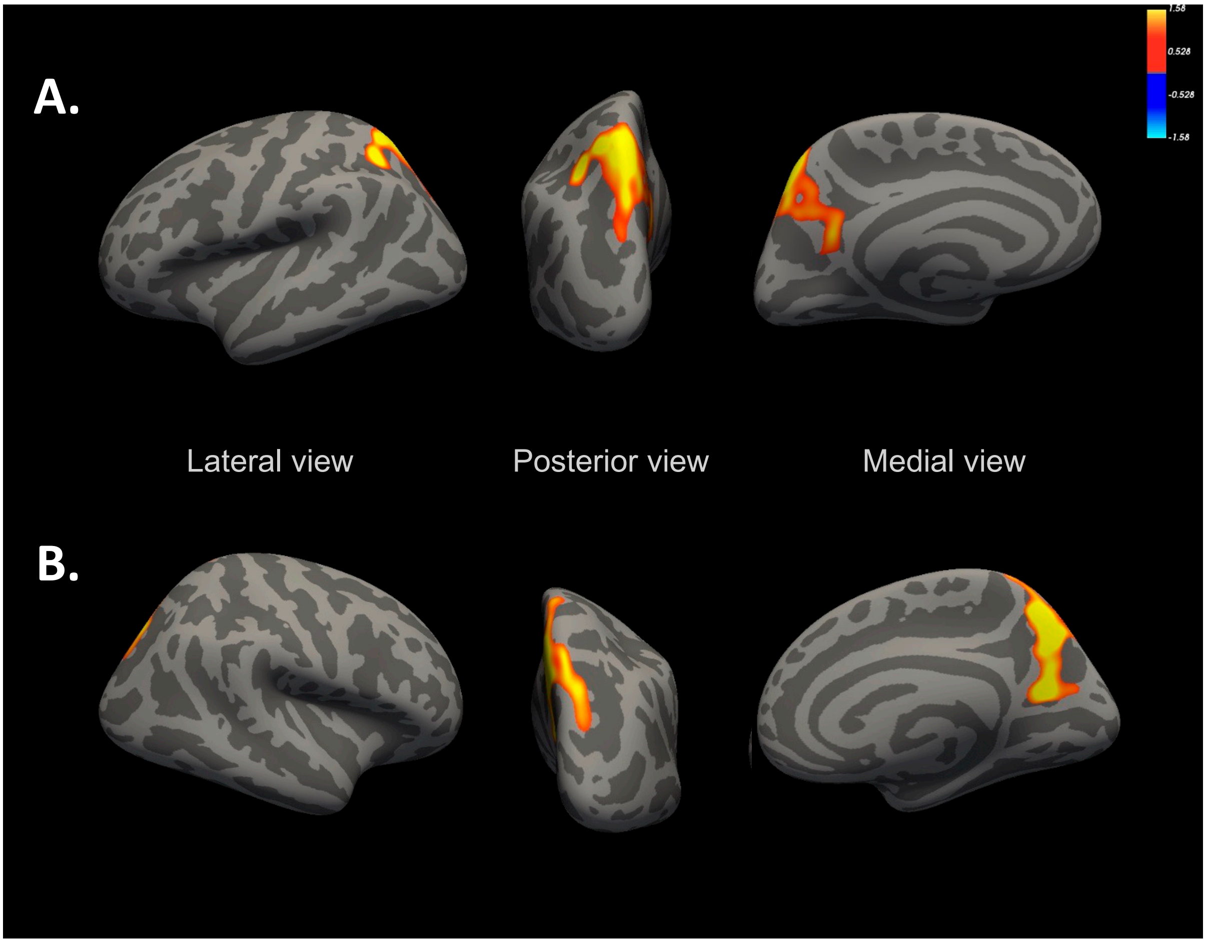

A. Left superior parietal area, **B.** Right precunes area.
Maternal brain surface on lateral, posterior and medial views.
The color bar indicates logarithmic scale of p values (-log10). Red to yellow color shows increase in the Mediterranean diet group participants compared to usual care group.
Images are generated from a general linear model with total intracranial volume, age, MR protocol and nulliparity, Mediterranean diet score at the initial assessment, socio-economic status and total energy intake at the final visit.

References

1. Juton, C.; Castro-Barquero, S.; Casas, R.; Freitas, T.; Ruiz-León, A.M.; Crovetto, F.; Domenech, M.; Crispi, F.; Vieta, E.; Gratacós, E.; et al. Reliability and Concurrent and Construct Validity of a Food Frequency Questionnaire for Pregnant Women at High Risk to Develop Fetal Growth Restriction. *Nutrients* **2021**, *13*, 1629, doi:10.3390/nu13051629.
